# Patient centered research to improve community involvement (PaRTICIpate) in diabetes self-management: a conference series for developing collaborations between researchers, stakeholders, and patients

**DOI:** 10.1186/s41687-018-0074-1

**Published:** 2018-10-24

**Authors:** Ashley S. Crumby, Erin R. Holmes, Meagen Rosenthal

**Affiliations:** 1 Department of Pharmacy Administration, University of Mississippi School of Pharmacy, University, Mississippi, Faser Hall 221, PO Box 1858, University, MS 38677 USA; 2 Department of Pharmacy Administration, University of Mississippi School of Pharmacy, University, Mississippi, Faser Hall 233, PO Box 1858, University, MS 38677 USA; 3 Department of Pharmacy Administration, University of Mississippi School of Pharmacy, University, Mississippi, Faser Hall 238, PO Box 1858, University, MS 38677 USA

**Keywords:** Patient-centered research, Diabetes, Community based participatory research, Patient engagement

## Abstract

**Background:**

A patient-centered approach to research development is important to the creation of research evidence that is meaningful and beneficial to patients. Collaboration between patients, stakeholders, and researchers, where patients serve an integral role in all aspects of the research development process, is integral to achieving these twin objectives.

**Results:**

This paper presents a unique approach to engaging patients and stakeholders in research by describing a conference series focused on meaningfully integrating patients in each phase of the project. Through three meeting phases, patients were not only introduced to patient-centered research (PCR) concepts, but they also led discussions about diabetes self-management and developed PCR questions. A total of 17 questions were developed represented by four main themes: communication, patient knowledge and perceptions, diabetes prevention, and diabetes management. Through patient feedback, three research questions were each identified as immediate priorities for development into research project proposals.

**Conclusion:**

To our knowledge, the use of a conference series designed to teach patients about research, encourage collaboration across stakeholder groups, and write research questions has not been described in the literature. Moreover, this approach has proven successful in recruiting and retaining patient participation through the life of the project. This project has also identified a number of issues for consideration by future researchers looking to meaningfully engage patients in the development of research proposals.

## Background

There is a growing body of research demonstrating a positive relationship between patient-centered care and improved health outcomes. However, there remains a gap between research evidence generation and its adoption by patients. According to the Patient-Centered Outcomes Research Institute (PCORI), one explanation for this gap is that traditional research is not created for application by patients, but through the preconceptions of clinicians and researchers [1, 2]. Patient-centered research (PCR) represents a collaborative effort wherein researchers and patients work together to generate mutually beneficial research evidence [2, 3]. Community-oriented approaches such as participatory action research and community-based participatory research have helped shift the role of the patient in the research process from subjects of research to stakeholders in research [4–6].

The inclusion of the patients’ perspective in the development of research has a number of potential benefits. One of the most important benefits of PCR to patients is the creation of research questions and evidence that are relevant, meaningful, and trusted by patients [1]. This can result in faster adoption of research findings due to the increased applicability of findings to patients’ everyday management of their conditions [1, 7, 8]. Another advantage is that patients feel listened to, valued, and proud of the ability to contribute to the research community [9]. Patients reported that engagement in the research process increased their confidence and feelings of self-worth, while also providing them with a sense of mutual support from fellow patients [9]. Finally, studies have shown that teaching patients about the research process increased general knowledge of research, as well as of specific studies, which leaves patients with a positive attitude toward research and increased trust in researchers [9].

Although PCR has many benefits, it can also present several practical challenges to research stakeholders including patients, researchers, and clinicians. A review of 47 PCORI projects outlined several challenges faced by patient-centered research projects including a lack of stakeholder time due to outside work and life commitments, lack of time for the research team to engage stakeholders meaningfully, and lack of stakeholder training and background in the research process [10]. Other important challenges mentioned included difficulty establishing trust and learning how to work together [10].

Recent interest in PCR and comparative effectiveness research (CER) has led to the need to develop more effective methods for engaging various stakeholder groups in research [2, 11, 12]. To begin, stakeholders are typically defined as, “individuals, organizations, or communities that have a direct interest in the process and outcomes of a project, research, or policy endeavor” [2], and can include patients, the public, purchasers, payers, policy makers, and principal investigators [3]. A review of the literature revealed a number of relevant studies discussing the stakeholder engagement process. One project identified three levels of engagement with stakeholders including communication, consultation, and participation [2]. Communication focused on researchers relaying information to stakeholders, while consultation focused on stakeholders conveying information to researchers [2]. The authors concluded by advocating for “participation” with a focus on the bi-directional flow of information between researchers and patients [2]. A second project presented an engagement approach utilizing the plan-do-study-act cycle [3]. Here the first step was to prioritize engagement and to adopt language in proposals reflecting recognition of stakeholders’ value in the research (i.e. plan) [3]. The second step involved testing how to integrate the stakeholders effectively (i.e. do) [3]. The third step was to evaluate these various integration approaches to see which are most successful (i.e. study) and the fourth step was to report the outcomes of these evaluations and implement changes in the future (i.e. act) [3].

A third project introduced a five-step approach to stakeholder engagement intended to identify and prioritize relevant diabetes care and prevention CER questions [13]. Step one involved an online survey sent to people with diabetes to identify important diabetes-related topics. In step two the survey results were presented at an in-person meeting of representatives from groups including federal agencies, advocacy organizations, disadvantaged populations, delivery systems, and patients for discussion and the development of a list of high-priority project ideas [13]. Step three involved the evaluation of the high-priority list by the research team to select the projects most suited for pilot study. Step four further narrowed down and specified this list to the top five CER pilot project concepts [13]. In step five this list of five project ideas was presented to the stakeholders from step two for feedback and the selection of three pilot projects [13]. This five-step approach yielded positive feedback from the stakeholders [13].

Each of these previous projects provides important insights into how to engage stakeholders in the research development process from a focus on the need for a bi-directional flow of information [2], to the need to continually evaluate the engagement process for improvement [3], and the need to check back in with stakeholders to ensure they feel their voices have been adequately captured [13]. However, taken individually these approaches do not address all of the important barriers to stakeholder engagement in PCR. In this paper, we outline another theoretically-based approach to stakeholder engagement intended to bring together important insights from previous works, while also addressing previously unaddressed barriers such as lack of stakeholder training in research [10]. Most importantly, this paper focuses on a process for how patients^1^ with Type 2 diabetes were meaningfully included in each phase of the project.

## Methods

### Project design

**Fig. 1:**
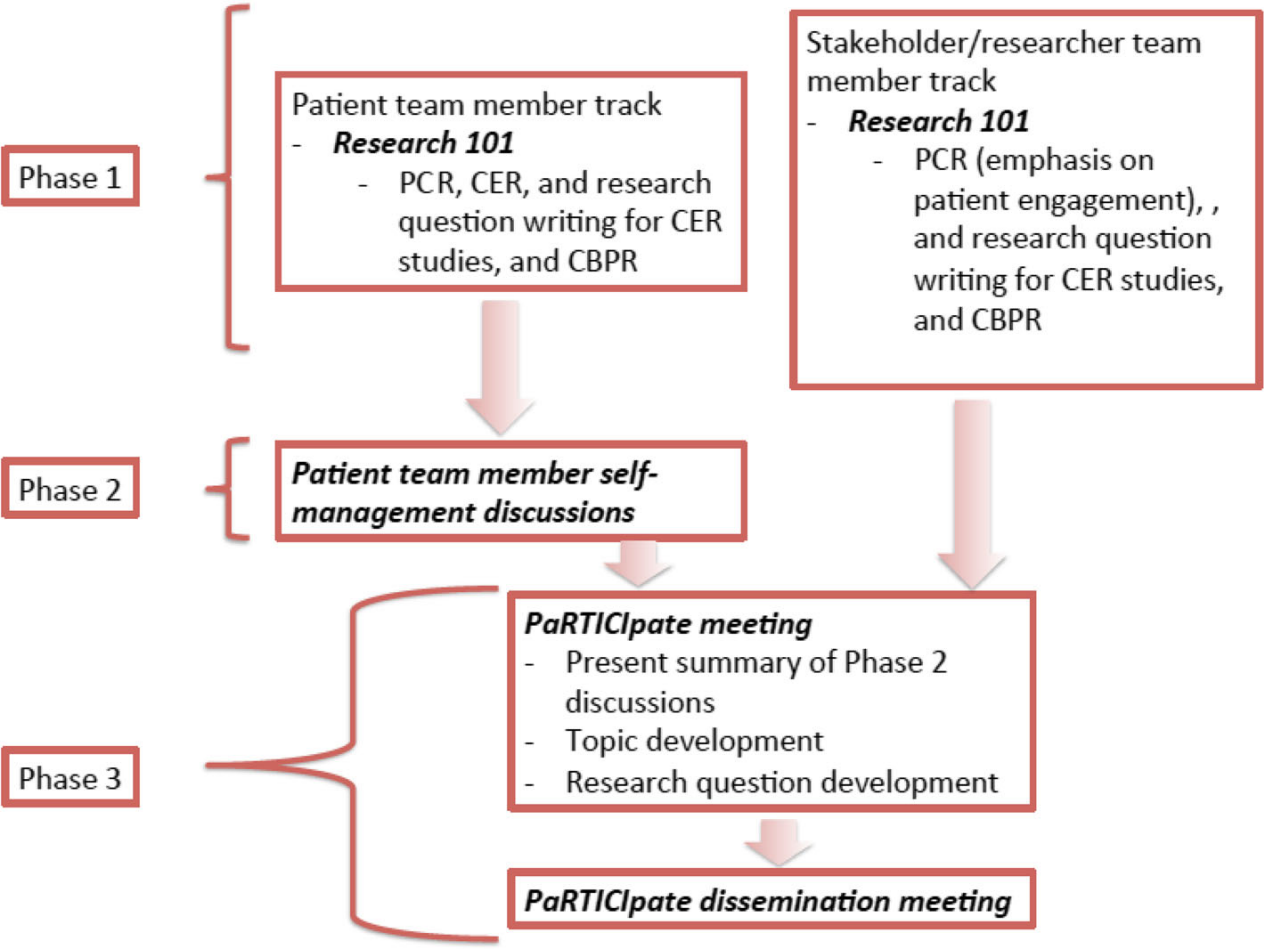
Conference series flow diagram

To achieve the objective of stakeholder engagement, this project utilized a conference series consisting of an early patient engagement consultation followed by three meeting phases (see Fig. 1). Early patient engagement involved meeting with five patients who self-reported a Type 2 diabetes diagnosis to obtain feedback on the underlying idea for the conference series. Phase 1 involved Research 101 meetings wherein both patients and stakeholders were introduced to the language of PCOR, CER, and research question development. Phase 2 consisted of diabetes self-management discussions among patient participants. Phase 3 comprised both the PaRTICIpate meeting, which brought together team members for the development of PCR questions, as well as a series of dissemination meetings that fed the research questions back to stakeholders for further consultation and discussion.

This project received a waiver of IRB approval. The Eugene Washington PCORI Engage Awards: Conference Support funding opportunity is specifically designed to support capacity building for future research, rather than to conduct research [14]. More specifically, this opportunity is intended to encourage the active integration of patients, caregivers, clinicians, and other healthcare stakeholders in the development of PCOR and CER research projects [14]. In line with this mission, and the objective of this proposed project, research was not being conducted, rather a partnership was forged to develop research questions for future research projects. As such, traditional routing through an IRB office was not necessary.

### Theoretical frameworks

This project design was based on two theoretical frameworks. The first framework was the community-based participatory research (CBPR) framework, which is defined as:

“A collaborative approach … designed to ensure and establish structures for participation by communities affected by the issue being studied, representatives of organizations, and researchers in all aspects of the research process to improve health and well-being …” [15].

In practice, this definition is operationalized through the application of four principles, beginning with the idea that the community itself is recognized as a unit of identity [15]. While the “community” traditionally referred to is a physical location, like a town, in this instance the “community” was people affected by Type 2 diabetes. The second CBPR principle is the idea of building on the community’s strengths [15]. As outlined in PCORI’s strategic plan, patients are the experts in their own conditions, and harnessing that experience creates knowledge that is relevant to those patients, and is therefore more likely to be adopted and used [1]. Moreover, beginning with a focus on the community’s strengths helps to improve the self-efficacy of patient collaborators by empowering them to rethink available resources [16]. The third CBPR principle encourages active collaboration across various stakeholder groups,^2^ and in all phases of the research development process [15]. In particular, this principle encourages all stakeholder groups to work together. However, before all participants can feel prepared to fully engage in these discussions the fourth, and final principle, of knowledge-sharing must be applied [15].

The second framework informing this projects’ design was the Indigenous consensus method, developed by Maar et al. (2015). This method, “…invites cultural values and knowledge gained from the lived experience of participants, by eliciting the perspectives of those who are eventually affected by the intervention …” [15]. Although similar to the intent of CBPR, the explicit mention of “cultural values” and “lived experiences of participants” reinforces the patient-centered focus by providing a specific space for the open discussion of these values and experiences by patients. Patient collaborators must feel comfortable and safe in discussing their own unique perspectives on living with Type 2 diabetes. Furthermore, this framework served as a reminder to researchers to listen carefully to patients before applying preconceived understandings of previous diabetes self-management literature. The Indigenous consensus method outlines a three-step process adapted for this project, which also mirrors the five-step plan outlined in the background [13]. The first consensus step presents group members with a set of key topics, usually generated by researchers, and asking them to identify an important sub-set of topics [15]. The second consensus step takes the identified sub-set of topics and considers important implementation issues, thereby revising the list based on the discussion [15]. The third, and final, consensus step takes the finalized list of topics from step two and develops CER questions for further study [15].

### Preliminary patient consultation

Since the community in question had been defined as patients who have Type 2 diabetes the next step in the development of the project was to reach out to those community members to apply the second CBPR principle of leveraging strengths. The early patient engagement consultation was conducted to allow for patient participation in the initial stages of the conferences series development. Five patients with Type 2 diabetes, two recruited by the director of a wellness center in Charleston, MS, and three recruited by those original two patients, were included. During this consultation, the proposed purpose of the conference series, along with a graphic outline of the phases of the meetings were presented (an earlier version of Fig. 1). Overall these patients were very interested in the proposed purpose saying that they were particularly interested in learning more about diabetes self-management.

They also provided five specific recommendations for how to move the conference series forward successfully. First, all patient participants said it would be very important to connect with a “central” community member, who is well connected and could help spread the word about the conference series. Second, with respect to participant compensation, they stated that issuing checks could jeopardize government benefits for elderly and low-income participants; therefore gift cards were recommended. Third, they suggested that some basic background information about participants be collected, including time since receiving diabetes diagnosis so presentations could be tailored to participants. Fourth, all presentations should be interactive, with a focus on aural presentations and visual aids, rather than written and printed material, to accommodate those with sight and literacy issues. Fifth, they suggested all meetings provide patients with some diabetes education and the opportunity to ask questions.

### Recruitment and team members

Using the first patient consultation recommendations, university-based team members worked with primary contacts in each community to get out the word about the conference series and the meetings. This was augmented by advertisements in local newspapers, distributing flyers to community establishments, and visiting and making announcements at local health-related events in each community to yield a diverse range of participants. As a result of these recruitment efforts, three groups of participants became involved in the conference series: Patient Team Members, Stakeholder/Researcher Team Members, and Key Personnel Team Members. The titles assigned to each of the groups were inspired by the terminology utilized by PCORI, and are intended to recognize the unique contributions of each group.

Patient Team Members were identified and recruited from three communities in northern Mississippi (Charleston, Oxford, and Saltillo), which stretch across the state from West to East. This particular area of Mississippi was selected for two reasons. The first, and most important reason was geographic proximity. Mississippi is a large rural state, and residents often face transportation barriers. For a PCR collaboration to be successful, it is important to foster relationships between team members wherein they will have the opportunity to meet face-to-face. The second reason for selecting these particular communities was that they represented a diverse population of patients (see Table 1). Charleston is located in a sparsely populated county, which has the highest proportion of African American people, and the highest percentage of residents below the poverty line [17]. Oxford, by contrast, is located in a county with a larger population and a lower percentage of African American residents, as well as fewer people below the poverty line [17]. Saltillo is located in the most densely populated county, and has a higher proportion African American people than the Oxford, but a lower proportion of people below the poverty line [17]. Interestingly the majority of participants from both Charleston and Oxford were African American, while the majority of participants from Saltillo were Caucasian.

**Table 1:** Demographics of participating communities^a^

Town/city	Charleston	Oxford	Saltillo
Population density^b^	23.8	75.0	184.3
% of population identified as African American	56.3%	23.8%	29.4%
% of population below the poverty line	32.9%	21%	17.4%

^a^All data presented in this table comes from the most recent census data available from the State of Mississippi [11]

^b^Measured as persons/square mile

This patient population also represented a diverse range of experience with diabetes. When looking at the group as a whole, several patients were relatively new to diabetes self-management having been diagnosed less than a year prior to our meetings, while other had been managing this condition for more than 20 years. Across the three communities, patients had been living with diabetes for an average of 8 years although this number differed across communities. Oxford, for example, had an average time since diagnosis of 8 years while the average time since diagnosis for the Saltillo community was 13 years. Charleston had the lowest average time since diagnosis of just three and a half years, representing the largest group of newly diagnosed patients participating in the project.

Stakeholder/Researcher Team Members consisted of various healthcare providers, and representatives from governmental and insurance organizations. Whenever possible Stakeholder/Researcher Team Members were identified and recruited from the same counties as patient participants, but depending on the nature of their work, some were recruited from other geographical areas. For instance, government representatives were invited from the state capital of Jackson. The final group of Stakeholder/Research Team Members consisted of six pharmacists, two nurses and diabetes educators, a nutritionist, a program administrator from the medical center in Jackson, a state director of a quality improvement and prevention organization, and two additional researchers from Oxford.

The Key Personnel Team consisted of seven university-based or affiliated people with various backgrounds. Four Key Personnel Team Members were healthcare providers including a pharmacist certified in diabetes management, as well as three members with advanced degrees in health services research. Of the remaining three members, two hold advanced degrees in health services research. One member is also the director of a local wellness center. The final member is the primary patient participant and advocate.

## Results

In the following sections the results of each of the three meeting phases from Research 101 through the dissemination meetings will be outlined.

### Phase 1: Research 101 meetings

The Research 101 meetings represent the application of the fourth CBPR principal, knowledge sharing to address inequalities [15]. These meetings established a common approach and language for all team members, and improved a sense collaboration and communication. In these meetings participants were introduced to the principles of CBPR and the Indigenous consensus method, PCOR, CER, and research question development with a special emphasis on how these approaches applied to improving self-management for people with Type 2 diabetes. To foster a feeling of comfort for team members, these meetings were held separately for Patient and Stakeholder/Researcher Team Members. The Patient Team Member the Research 101 meetings were held in local and accessible venues for two reasons. First, this reduced travel burden for community members, making it easier for them to attend. Second, the Key Personnel Team Members wanted to demonstrate their investment in the process by meeting team members in their own communities. The Stakeholder/Research Team Member meetings were held, in Oxford and Jackson.

Based on recommendations four and five of the preliminary patient consultation, the meetings involved interactive sessions wherein Patient Team Members could ask questions about diabetes self-management, as well as practice creating research questions using the PCORI question writing guidelines [18]. A local diabetes expert, and Key Personnel Team Member, was available to questions related to the treatment and management of Type 2 diabetes. It was also at this meeting where initial background information was collected following patient consultation recommendation three. The patient Research 101 meetings were well attended, exceeding the projected participation levels of 10 total participants in two of the three communities. There were 19 people at the Charleston meeting, 18 people at the Oxford meeting, and 10 people at the Saltillo meeting. Patient Team Members’ discussion focused on improving general knowledge about diabetes management, diet, diabetes prevention, medication and treatment options, and diabetes complications.

The Research 101 Stakeholder/Researcher Team Member meetings were less well attended than the patient meetings. At the Oxford meeting, there were three stakeholder participants, and at the Jackson meeting there were seven stakeholder participants. Unlike the Patient Team Member meetings, which were unstructured and driven by the participants questions and concerns, the stakeholder meetings began with a brief video interview with a Patient Team Member, wherein she shared her personal diabetes story. A discussion then followed regarding strategies for patient engagement, because patient engagement in research development is largely new to both researchers and clinicians [1]. This was then followed by a more detailed discussion of PCOR, CER, and research question development.

Following these meetings, the Patient Team Members were asked to complete a satisfaction survey to improve future interactions. Overall, the response was positive with an average satisfaction score of 6.4 on a 7-point scale. Participants reported enjoying the ability to share their experiences and struggles with other people who also have diabetes. Some Patient Team Members even indicated it was the first time they had ever taken part in a discussion of their condition. One participant did indicate a dislike for the research proposal process, but overall participants planned to continue with the project.

### Phase 2: Patient team member discussion meetings

Patient Team Members discussion meetings helped to identify key factors in patients’ attitudes towards and use of self-management in their daily lives. This group of meetings was designed to extend the patient-centered focus of this project and represents the first adapted step in the Indigenous consensus method [15]. As previously stated, research teams often developed lists of key topics and presented them to participants [13, 15]. However, this work sought to preserve the cultural values, lived experiences, and language, of Patient Team Members, unlike past self-management research, which has focused on medical/behavioral management [16]. Patient Team Members were asked to talk about their perceptions of “self-management,” daily self-management activities, their level of satisfaction with these activities, what other information they may need, and their confidence in implementing new activities. These discussions gave Patient Team Members a protected opportunity to think about and discuss self-management.

Two discussion meetings took place in each community and were led by two members of the Key Personnel Team. Each of the original Patient Team Members from the Research 101 meetings was asked to identify one family member, caregiver, or friend with Type 2 diabetes or pre-diabetes and invite them to participate. This increased the diversity of experiences and directly addressed the first recommendation of the patient consultation to leverage community members to spread the word about the project. These discussions were also well attended. Charleston had 17 participants across the two meetings, Oxford had 28, and Saltillo had 10. A summary of key factors from these discussions was then developed, and circulated to all Patient Team Members participants for review and comment.

### Phase 3: PaRTICIpate and dissemination meetings

This meeting followed steps two and three of the Indigenous consensus method and allowed participants the opportunity to come together to discuss and ultimately decide on the topics that were particularly important to them [8]. During this meeting, participants interacted with people from other communities and shared ideas about diabetes self-management through carefully considered seating arrangements that mixed all team members in groups of 8–10 from across the communities and ensured that patients were always the largest group at the table. The day included a presentation of the discussion meeting summaries, an overview of the main topics that were identified during those meetings, and two breakout sessions. In the first breakout session groups were asked to consider the discussion meeting summaries and add any missed topics. In the second breakout session groups were asked to narrow down the list of topics and design the research questions using the PCORI guide to writing research questions [18]. Each small group was guided by a facilitator and presented their ideas to the large group at the end of the second breakout session. In addition to these opportunities for discussion, participants were also provided some education about facts and myths around diabetes management, as well as some nutrition tips. A total of 33 Patient Team Members, 11 Stakeholder Team Members, and 4 Researcher Team Members attended the PaRTICIpate meeting.

A total of 17 research questions were developed by the patients at the PaRTICIpate meeting, and were divided into four main themes:

1. Communication – this topic includes ideas about communication from both the provider to the patient as well as from the patient to the provider. Example: “Can we develop a training program to better prepare patients for meetings with healthcare providers?”
2. Patient knowledge and perceptions – this topic focuses on how patients’ and family members’ level of diabetes knowledge impacts how they understand the disease and manage the condition. Example: “How does family history of diabetes affect a patient’s diabetes knowledge and motivation for self-care?”
3. Diabetes prevention – this topic focuses on how we can prevent people from getting diabetes or how to prevent the progression from pre-diabetes to diabetes. Example: “How do we improve communication about pre-diabetes?”
4. Diabetes management – this topic focuses on how we can help people better manage their condition by giving them information they need and can readily use. Example: “How can we manage diet and weight in children with Type 2 diabetes?”

Following the PaRTICIpate meeting, attendees were again asked to provide feedback. Participants reported their favorite part of the meeting was the ability to come together in a group setting to discuss diabetes topics. They enjoyed the breakout groups and the ability to interact with each other and allow ideas to be shared between the different communities, particularly because they would not have been given an opportunity to interact otherwise.

Two rounds of dissemination meetings in each of our partner communities were then held. In the first round of these meetings all Patient Team Members were given the opportunity to read and review the 17 research questions that were developed at the PaRTICIpate meeting. Patients and caregivers were then asked to identify the three research questions they considered to be the most important. The most rated questions were then given priority for further development into research project proposals.

The first project, “A Patient Decision Aid to Improve Diabetes Self-Management in the Community Pharmacy Setting”, focuses on the creating of a tool that can help patients with Type 2 diabetes to identify knowledge gaps and prioritize self-management support. The second project, “Patient Perceptions of and Attitudes Toward a Weight Management Program Offered by Community Pharmacists”, involves the collaborative development of a community pharmacy weight management program. The third project is entitled “Perceptions of Type 2 Diabetes Among Those with Family History of the Disease” and will focus on how family members talk about diabetes self-management among themselves in an effort to better understand how this communication affects self-management practices. These proposals were reviewed and commented upon by Patient Team Members in the second round of dissemination meetings. In the mean time the proposal, “Patient Perceptions of and Attitudes Toward a Weight Management Program Offered by Community Pharmacists” has received funding from the Mississippi Center for Clinical and Translational Research Pilot Project Program, which is supported by the National Institute of General Medical Sciences of the National Institutes of Health under Award Number 1U54GM115428.

## Discussion

This project provides one approach for helping patients, stakeholders, and researchers come together in meaningful collaboration to develop PCR research questions. The application of CBPR and the Indigenous consensus frameworks provided a step-wise approach for achieving PCR by reinforcing the need to adequately account for the perspective of effected communities and led to the development of 17 research questions for future investigation [1]. Reflection on this process through the satisfaction surveys completed by Patient Team Members and constant reflection by Key Personnel Team Members also revealed a number of important lessons for future engagement projects. Lesson one was the importance of not creating too rigid of a schedule for meetings and being flexible with expectations. Most Patient Team Members were not accustomed to traditional didactic settings and also had important needs they felt participation in this conference series could meet. Key Personnel Team Members had to take their cue from Patient Team Members in all circumstances.

Lesson two was that even relatively proximal communities can be very different from each other. This difference was most notable with regard to time since diagnosis. In particular, those who had been living with diabetes longer naturally had more experience and were also more willing to provide advice and share their stories. However, it was interesting to note that these patients also had struggles that mirrored those of newly diagnoses patients, especially in relation to things like food choices. How and what to eat was a consistent struggle for all of our Patient Team Members. Lesson three was that logistics play a vital role in ongoing participation and the development of trust within the communities. For example, transportation was identified as a significant potential barrier for participation in the PaRTICIpate meeting. As such, free transportation was provided for all who identified the need in each of our partner communities. This step ensured a diverse group of participants and a rich discussion.

Lesson four, and the most important lesson for the Key Personnel Team Members, was just how interested the patients are in participating in this kind of work. They eagerly shared their honest experiences and thoughts, asking deeply personal, and potentially sensitive, questions with the desire to learn about their condition. This added to the richness of the discussions, and let Key Personnel Team Members know we had gained patients’ trust. Moreover, these revelations also demonstrated patients’ need for tailored education they could apply in their daily lives.

Lesson five was that attracting stakeholders for this research proved to be more difficult than originally anticipated. As has been discussed many efforts were made to accommodate patients’ needs, however, Key Personnel Team Members, wrongly assumed that stakeholders would inherently appreciate the value of this work and sacrifice and volunteer their time. In retrospect, this was not a valid assumption and going forward more careful consideration of the needs of stakeholders could include opportunities for one-on-one telephone interviews rather than group meetings. This flexibility would enable discussions to be worked into normal daily workflow rather than committing to after work meetings.

The objective of this project was to thoughtfully integrate patient stakeholder voices into every step of the research development process, learning from previous work in this area. To this end this project achieved a bi-directional flow of information between Patient and Key Personnel Team Members [2], continually evaluated the engagement process by asking Patient Team Members directly or reflecting on observations by Key Personnel Team Members [3], and checked in with stakeholders to make final decisions on future research projects [13]. This work also focused on obtaining the voices of patients by collecting ideas about diabetes self-management directly from Patient Team Members, rather than using a survey or list of pre-established ideas [13, 15]. Finally, after careful reflection on the process of the conference series five lessons that future investigators can apply to their own stakeholder engagement projects were identified and outlined.

## Conclusions

Overall, the aim of generating patient-centered diabetes self-management research questions was achieved using the steps outlined herein. The lessons taken from this experience will be valuable to other researchers hoping to develop their own PCR questions. Including patients in the research process and meaningfully collaborating with them fundamentally changed how Key Personnel Team Members approach the research development process. Through a partnership between patients, stakeholders, and researchers the possibility for improvement in self-management of diabetes is encouraging and should be considered the goal for future research initiatives.

## Abbreviations

CBPR: Community-based practice research
CER: comparative effectiveness research
PCR: Patient centered research
PCORI: Patient-Centered Outcomes Research Institute

## Footnotes

1 Using the definition of patients offered by PCORI’s strategic plan, patients were considered as being any individual who either has lived experience with Type 2 diabetes, or a person who lives with the condition indirectly, such as a caregiver, spouse, child, or partner (20).

2 Stakeholders, for the purposes of this project, will include clinicians (i.e., physicians, nurses, pharmacists), purchasers, policy makers, and researchers (20).
